# A crystal-structural study of Pauling–Corey rippled sheets†‡

**DOI:** 10.1039/d1sc05731f

**Published:** 2021-12-08

**Authors:** Ariel J. Kuhn, Beatriz Ehlke, Timothy C. Johnstone, Scott R. J. Oliver, Jevgenij A. Raskatov

**Affiliations:** Dept. of Chemistry and Biochemistry, UCSC 1156 High Street Santa Cruz California USA jraskato@ucsc.edu

## Abstract

Following the seminal theoretical work on the pleated β-sheet published by Pauling and Corey in 1951, the rippled β-sheet was hypothesized by the same authors in 1953. In the pleated β-sheet the interacting β-strands have the same chirality, whereas in the rippled β-sheet the interacting β-strands are mirror-images. Unlike with the pleated β-sheet that is now common textbook knowledge, the rippled β-sheet has been much slower to evolve. Much of the experimental work on rippled sheets came from groups that study aggregating racemic peptide systems over the course of the past decade. This includes MAX1/DMAX hydrogels (Schneider), L/D-KFE8 aggregating systems (Nilsson), and racemic Amyloid β mixtures (Raskatov). Whether a racemic peptide mixture is “ripple-genic” (*i.e.*, whether it forms a rippled sheet) or “pleat-genic” (*i.e.*, whether it forms a pleated sheet) is likely governed by a complex interplay of thermodynamic and kinetic effects. Structural insights into rippled sheets remain limited to only a very few studies that combined sparse experimental structural constraints with molecular modeling. Crystal structures of rippled sheets are needed so we can rationally design rippled sheet architectures. Here we report a high-resolution crystal structure, in which (l,l,l)-triphenylalanine and (d,d,d)-triphenylalanine form dimeric antiparallel rippled sheets, which pack into herringbone layer structures. The arrangements of the tripeptides and their mirror-images in the individual dimers were in excellent agreement with the theoretical predictions by Pauling and Corey. A subsequent mining of the PDB identified three orphaned rippled sheets among racemic protein crystal structures.

## Abstract 2

Following the seminal theoretical work on the pleated β-sheet published by Pauling and Corey in 1951, the rippled β-sheet was hypothesized by the same authors in 1953.

## Graphical abstract

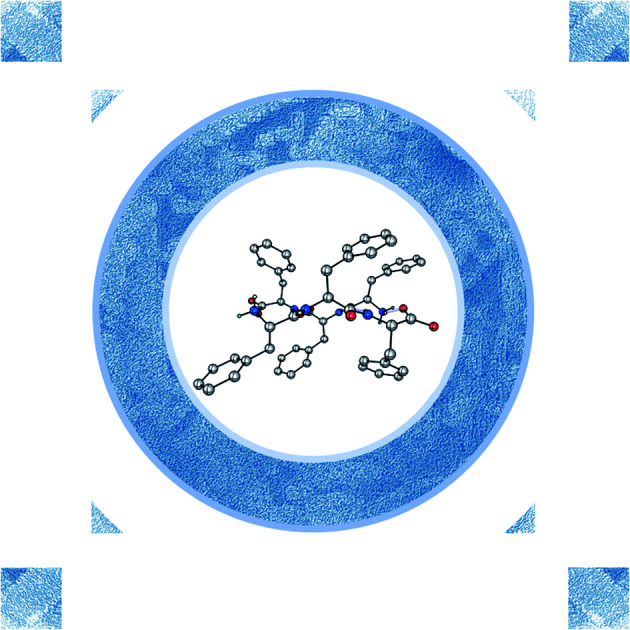

## Introduction

Peptides with mixed chirality may be used to access frameworks with unique properties, including protease-resistant peptide drugs,^1,2^ hydrogels with enhanced rigidity,^3,4^ aggregation blockers,^5,6^ amyloid oligomer-to-fibril converters,^7,8^ and mechanistic tools.^9,10^ Mirror-image proteins may also be used to enhance crystallization of proteins that are hard to crystallize, sometimes by creating unique interactions between the protein enantiomers.^11–14^ A systematic incorporation of d-amino acids into proteins and peptides is expected to give access to a huge structure–function space that cannot be accessed in any other way.

**Fig. 1:**
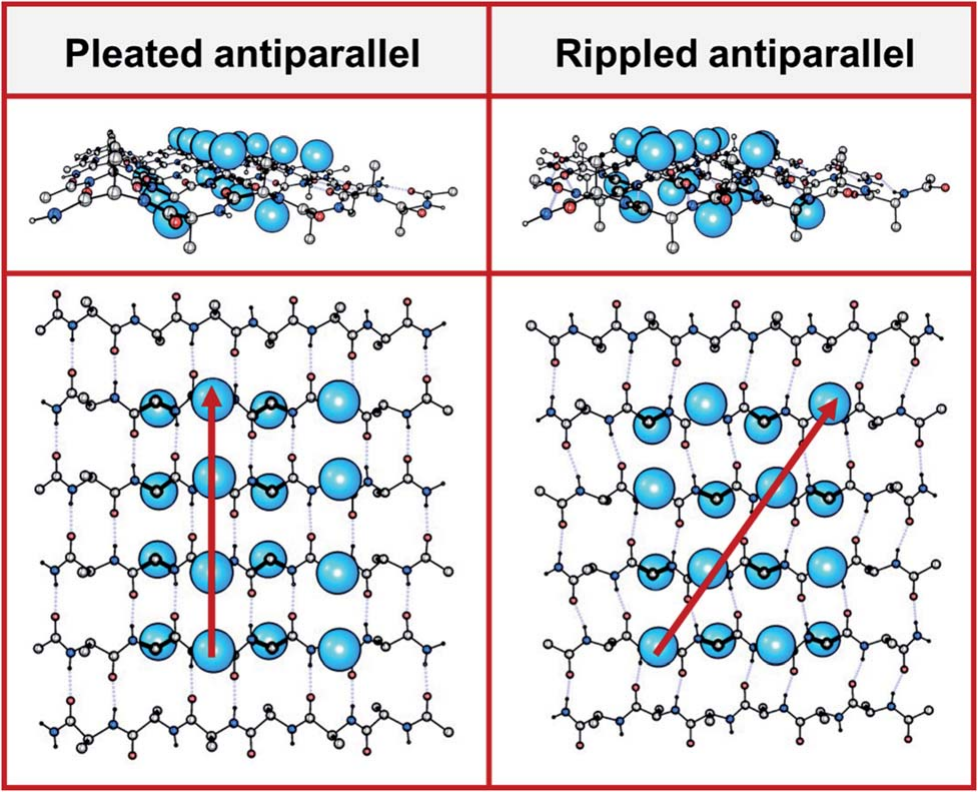
Left panel: antiparallel pleated sheet in different projections. Right panel: antiparallel rippled sheet in different projections. A selected number of amino acid side chains are depicted as blue spheres on the left panel (pleated, along red vertical line) and on the right (rippled, along red diagonal).

In 1951, Pauling and Corey introduced the pleated β-sheet as a two-dimensional periodic layer configuration built from extended homochiral peptide strands.^15^ The pleated β-sheet rapidly established itself as a key protein structural motif that is commonly known in textbooks as the β-sheet. Thousands of protein structures have been published that contain β-sheets. This includes structures that may be as huge as a periodic, fibrillary β-sheet network on the one side and as small as a β-sheet dimer in the context of a globular protein on the other side. In 1953, Pauling and Corey introduced the rippled β-sheet as a configuration closely related to the pleated β-sheet, but with every alternate peptide chain mirrored, thus giving rise to unique structures.^16^ Some of the key structural differences between pleated and rippled β-sheets, including differences in hydrogen bonding and relative side-chain disposition in the β-sheet frameworks, have been discussed very recently.^17^ As illustrated in Fig. 1, in an antiparallel pleated sheet, amino acid side chains are aligned in a vertical line orthogonal to the peptide backbones (Fig. 1, left panel). In contrast, in an antiparallel rippled sheet, to reduce steric repulsion between the alternating enantiomeric peptides, the side chains are oriented diagonally across the peptidic network (Fig. 1, right panel).

Unlike with the pleated β-sheet (now known as the β-sheet), the growth of our body of knowledge on the rippled β-sheet has been extremely sluggish. The first experimental observation of an (antiparallel) rippled sheet was made in the 1970s by Lotz, Moore and Krimm, on polyglycine I.^18–20^ The authors used space group considerations to conclude that polyglycine I crystals contained rippled rather than pleated antiparallel sheets (monoclinic rather than orthorhombic unit cell geometry). Some three decades later, Lahav and co-workers used clever labeling strategies in conjunction with mass-spectrometry, to produce evidence for rippled sheet formation, based on templated peptide replication.^21,22^ Conversely, Chung and Nowick noted in their solution-phase NMR studies a thermodynamic preference for a dimeric pleated β-sheet, with the alternative rippled sheet observed as a minor diastereomer.^23^ A more recent study by Liu and Gellman is broadly consistent with Chung and Nowick.^24^ Our understanding of the interplay of thermodynamics and kinetics that underlie the formation of pleated *vs.* rippled sheets remains extremely limited. Experiments performed in the laboratories of Schneider,^3,4^ Nilsson,^25,26^ Raskatov,^7,8^ and Torbeev,^27^ showed that mirror-image peptide strands may assemble into rippled sheets, but there is also evidence that some sequences may favor homochiral association.^28^ The structural insights available for the MAX1/DMAX systems,^3^ a short Amyloid-β (Aβ) segment,^25^ and, most recently, racemic full-length Aβ40 (ref. 29) were obtained from theoretical calculations constrained by a fairly limited number of experimental data. These studies provide valuable insights into rippled sheets, but not experimental high-resolution structures.

It is interesting to note that not all racemic peptide mixtures form rippled sheets,^23,24,30^ as self-sorting into pleated sheets may also occur.^30^ We are just beginning to learn why some racemic peptide mixtures form rippled sheets (*i.e.*, are “ripple-genic”), whereas others prefer to form pleated sheets instead (*i.e.*, are “pleat-genic”). To systematically map out the structure–function space and to close this major knowledge gap, the field urgently needs high-resolution structures of rippled sheets. Here we report the X-ray crystal structure of (l,l,l)-triphenylalanine that is hydrogen-bonded to (d,d,d)-triphenylalanine in a dimeric antiparallel rippled sheet. We then draw comparisons with hitherto orphaned rippled sheet crystal structures that we discovered by searching the PDB for racemic proteins.

## Results

### Choice of system

The significance of the oligomeric phenylalanine motif for amyloid formation is well-established. For example, it is known that the hydrophobic LVFFA segment that spans the amino acid residues 17–21 of the Amyloid β (*i.e.*, Aβ17–21) peptide is crucial for Aβ fibrillization.^31^ Furthermore, Kiessling and coworkers have taken advantage of this by using the KLVFF segment for molecular recognition studies with Aβ.^31,32^ Reductionist studies of Aβ by Gazit and co-workers demonstrated that the short diphenylalanine peptide is itself capable of forming amyloid nanostructures.^33^ Unlike the dipeptide, FF, which has been shown to form water-filled nanovesicles and hollow tubes, the tripeptide, FFF, spontaneously assembles into a diverse set of supramolecular assemblies depending on conditions, such as solid nanospheres, nanorods, helical-ribbons, plates, dendrimers, and doughnuts,^34–36^ similar to what has been reported for Aβ,^37^ making it an interesting candidate from the standpoint of rippled sheet design. Additionally, Gazit and coworkers found that FFF demonstrated improved stability and peptide-network propensity over FF.^36^ The authors also reported Thioflavin T (ThT) positivity for the FFF assemblies, indicative of ordered β-sheet content.^36^

More recently, Nilsson and co-workers demonstrated that the Aβ16–22 segment, KLVFFAE, rapidly formed precipitates when mixed with its mirror-image counterpart klvffae, which the authors ascribed to rippled sheet formation based on isotope-edited FT-ICR mass spectrometric and solid state NMR spectroscopic experiments.^25^

Peptides containing bulky, hydrophobic amino acids Phe (F), Val (V), Ile (I) and Leu (L) are believed to be particularly prone to forming rippled sheets.^17^ Phenylalanine stands out because of its relative rigidity, which should favor crystallization.^38^ We chose a racemic mixture of (l,l,l)-triphenylalanine and (d,d,d)-triphenylalanine (*i.e.*, FFF:fff), as our model. The N- and C-termini of FFF and fff were kept as free amines and free carboxylates, respectively, to afford peptides that (a) are water-soluble and (b) favor a defined antiparallel arrangement due to coulombic attraction. Peptides were made on solid support and purified using a procedure similar to one we previously developed for Aβ purification (Fig. S1 and S2‡).^39^

### The FFF:fff dimer structure

Combination of concentrated solutions of FFF and fff led to rapid formation of a fine precipitate. Optimization of conditions led to a protocol, in which controlled cooling of a solution saturated with a racemic mixture of FFF and fff from 75 to 25 °C at a rate of 0.1 °C min^−1^ afforded single-crystal needles with length exceeding 3 mm. A short needle, suitable for single crystal X-ray diffraction was selected and the metric symmetry and Laue symmetry of the diffraction pattern obtained with Cu K_α_ radiation revealed that the crystal belonged to the monoclinic crystal system. Strict observance of Friedel's law and the 〈*E*^2^ – 1〉 value of 1.008 indicate that the crystal is centrosymmetric, suggesting that the molecules had crystallized as the racemic compound. Centrosymmetry was confirmed by analysis of the systematic absences, which unambiguously confirmed the space group to be *P*2_1_/*c*. The structure was solved using intrinsic phasing and refined against 0.84 Å-resolution data (Table S1‡). The resolution and quality of the data permitted anisotropic refinement of all non-H atoms and semi-free refinement of H-atom positions.

The asymmetric unit contains a single tripeptide in its zwitterionic form (Fig. S3‡). Both amides assume the expected *trans* configuration. The *ψ* angles of 114.6(2)° and 132.3(1)° and the *φ* angles of −124.7(4)° and −155.1(1)° for FFF fall within the range typically observed for β-pleated sheets. The side chains of the three residues assume, from N to C terminus, the *gauche*^+^ (*χ*_1_ = −63.2(2)°), *trans* (*χ*_1_ = −175.1(1)°), and *gauche*^−^ (*χ*_1_ = 70.7(2)°) configurations.

**Fig. 2:**
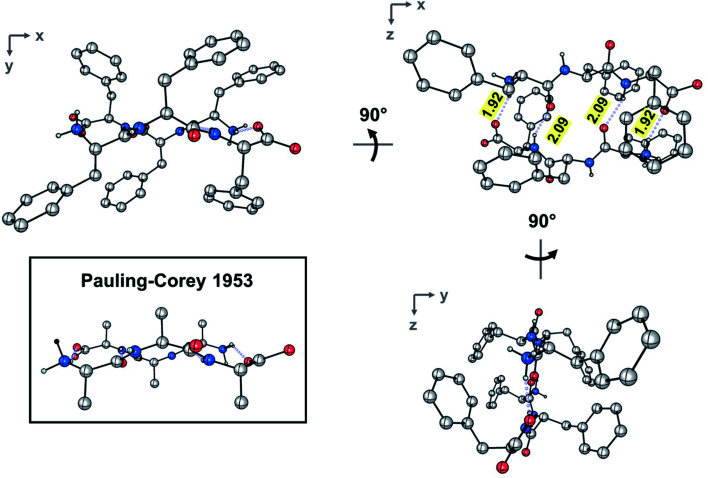
Ball-and-stick depiction of the experimental rippled antiparallel FFF:fff cross-β dimer, shown in three orthogonal projections. The Pauling–Corey rippled antiparallel backbone dimer is shown in the inset, with apical carbon atoms added geometrically to facilitate comparison; (color code: C, gray; O, red; N, blue).

The dimer resides on a crystallographic inversion center, across which FFF and fff form two symmetry-related pairs of hydrogen bonds (Fig. 2). The terminal ammonium and carboxylate groups form a salt bridge with a N⋯O distance of 2.7660(18) Å and a N–H⋯O angle of 152.8(19)°. The hydrogen bond formed between the neutral amide units features an expectedly longer N⋯O distance of 2.9097(18) Å and a N–H⋯O angle of 157.4(17)°. The hydrogen bonds comprise the only significant intermolecular contacts between the components of the dimer; the torsion angles assumed by each of the phenylalanine units allow them to effectively interleave given the inversion symmetry relating the two molecules. This arrangement of hydrogen bonds is in excellent agreement with the model put forward by Pauling and Corey (Fig. 2 and S4‡). In that original work, they model the antiparallel rippled sheet using a translation of 7.00 Å, which agrees well with the C_α,1_⋯C_α,3_ distance of 6.888(2) Å in the present crystal structure.

### Crystal lattice analysis

The crystal is held together by a combination of interdimer hydrogen bonds, ionic interactions, and van der Waals interactions (Fig. S5‡). In addition to interacting with the terminal carboxylate of the inversion-generated dimer mate, the terminal ammonium also forms hydrogen bonds to a glide-generated carbonyl of an enantiomeric tripeptide molecule (N⋯O = 2.7244(17) Å) and to the screw-generated terminal carboxylate of a molecule of identical handedness (N⋯O = 2.6645(18) Å). The internal amide N–H unit that is not involved in the antiparallel cross-β FFF:fff dimer also hydrogen bonds to this same screw-generated terminal carboxylate (N⋯O = 3.0168(17) Å). The H-atom positions in the final model are consistent with this hydrogen bonding pattern.

**Fig. 3:**
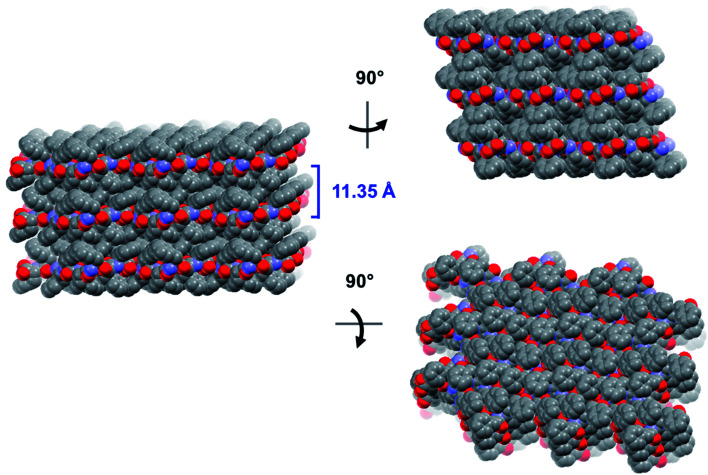
Long-range packing of the FFF:fff lattice, shown in three orthogonal projections. The layer-to-layer distance is indicated in blue.

**Fig. 4:**
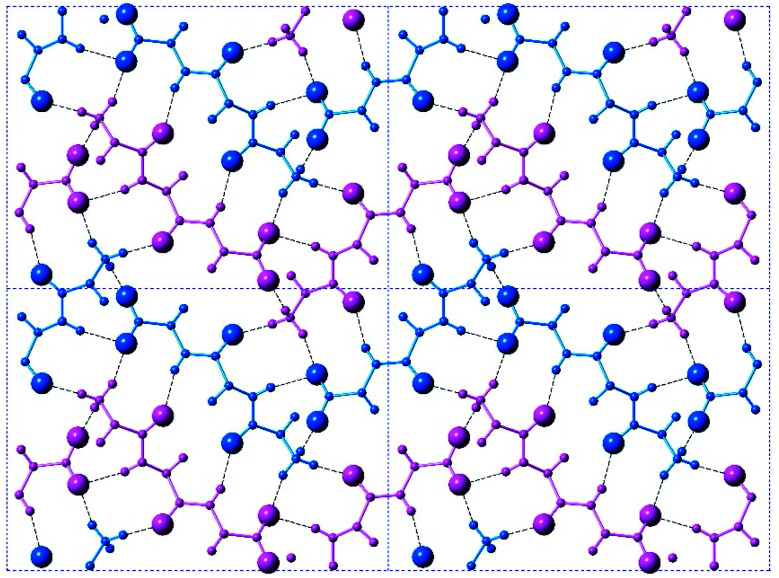
A top-on view of a single layer containing the peptidic backbones. Individual rippled antiparallel FFF:fff cross-β dimers are centered about the unit cell corners and center. l-peptides are shown in purple, and d-peptides are shown in blue.

These hydrogen bonds extend to form sheets parallel to the crystallographic *bc* plane (Fig. 3). These sheets feature a hydrophilic core bounded on both sides by hydrophobic layers. The layers stack on one another with an interlayer spacing corresponding to the crystallographic *a* lattice parameter of 11.3563(5) Å (Fig. S6‡). This nanoscale architecture, with clear alternation between hydrophobic and hydrophilic layers, is reminiscent of a phase separation. The dimeric rippled sheets do not assemble into extended “fibrillary” rippled sheets with long-range order, packing into a classic herringbone pattern instead (Fig. 4).

To confirm that an isolated FFF:fff rippled antiparallel cross-β dimer is in itself a stable arrangement, the dimer was subjected to full geometry optimization using Density Functional Theory (DFT) methods. The optimization produced only marginal local structural changes (Fig. S7 and Table S2‡), confirming that the structural features of the dimer are inherent to the β-rippled-sheet hydrogen bonding pattern and not crystal packing forces. This result stands in good agreement with our previous computational work on related rippled interfaces.^8,17,40,41^

## Discussion

**Fig. 5:**
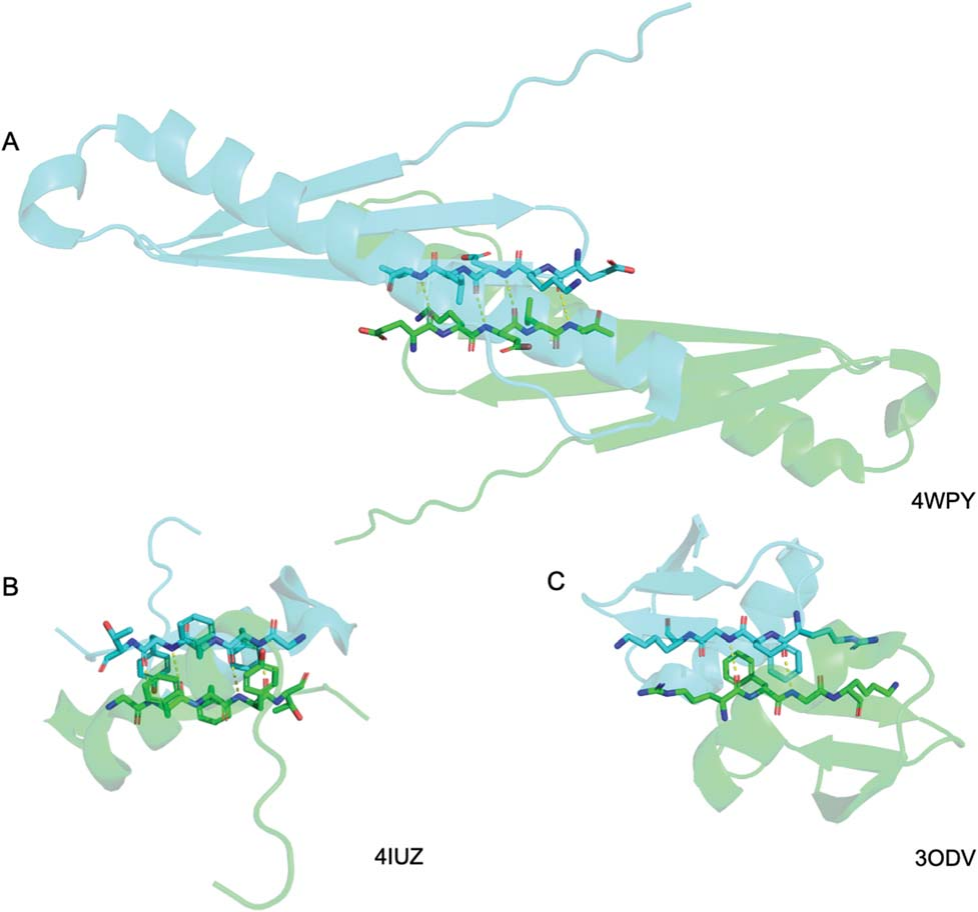
Detail of the antiparallel rippled motifs in the proteins selected by the PDB structural database mining. (A) Glu–lys–glu–leu–val sequence in RV1738.^42^ (B) Phe–phe–tyr sequence in ester insulin.^43^ (C) Lys–gly–phe–arg sequence in Kaliotoxin.^44^ PDB codes are displayed on the bottom right.

**Fig. 6:**
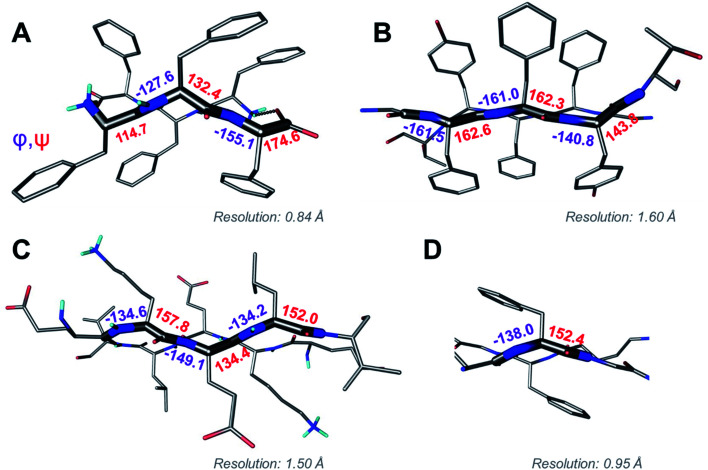
Ramachandran angle analysis for the rippled sheets noted with (A) the FFF:fff system; (B) racemic Ester Insulin (4IUZ);^43^ (C) racemic RV1738 (4WPY);^42^ (D) racemic Kaliotoxin (3ODV).^44^

Above we presented a range of structural features we were able to glean from a crystal-structural analysis of the FFF:fff lattice. To the best of our knowledge, this is the first time that a rippled sheet crystal structure is being discussed in the literature. However, owing to the efforts of racemic protein crystallography, many crystal structures that contain potentially interacting mirror-image protein pairs are now available. It seemed plausible that the enantiomers in some of those structures might interact *via* rippled sheets. We interrogated this possibility by searching the Cambridge Structural Database (CSD) and the Protein Data Bank (PDB), as described in the Materials and methods section. The CSD search revealed no rippled sheet structures. The PDB search identified three racemic protein crystal structures with a qualitative appearance suggesting the presence of antiparallel rippled sheets. We analyzed the three structures and validated that dimeric rippled sheets were indeed present in all three cases (Fig. 5). As such, we found that in the racemic crystal structure of the Rv1738 protein, the protein enantiomers interact through an antiparallel rippled sheet formed by the Lys–Glu–Leu triad and its enantiomer (Fig. 5A).^42^ We also found that, in the racemic ester insulin crystal structure, the enantiomers are bridged by a rippled sheet formed between the Phe–Phe–Tyr triad and its enantiomer (Fig. 5B).^43^ Finally, we observed a very short rippled sheet segment of only one Phe residue and its enantiomer in the racemic crystal structure of kaliotoxin (Fig. 5C).^44^ Whereas in those three structural studies, the authors did recognize there were mirror-image interactions between their protein pairs, none of them identified those interactions as rippled sheets, which may be why those important structural insights appear to have escaped the attention of the rippled sheet community thus far. To gain deeper insights into the backbone conformations associated with the four rippled antiparallel sheet structures, we analyzed their Ramachandran angles (Fig. 6). We noted that three of the rippled sheets contain internal L–Phe:D–Phe pairs, *i.e.*, (F:f). Their Ramachandran angles range from *φ* = −127.6° and *ψ* = 132.4° with FFF:fff (Fig. 6A) to *φ* = −161.0° and *ψ* = 162.3° with racemic ester insulin (Fig. 6B). This means that there is significant flexibility that is available to the (F:f) pair in the context of the antiparallel rippled sheet, which may become a useful design element if the interest of the materials community to the rippled sheet motif continues to grow.

Pleated β-sheets are often observed in fibrils formed by aggregating enantiopure peptides, where they tend to display a one-dimensional long-range order. Numerous structures are available through the work of the Eisenberg lab on steric zippers and related systems.^45–50^ Some examples are shown in Fig. S8.‡ In contrast to the long-range packing noted in the Eisenberg systems, we observed dimeric antiparallel rippled sheets with FFF:fff (Fig. 2), but those dimers did not form extended rippled sheets (Fig. 3 and 4). The lack of extended sheets may also be rooted in the hydrophobicity of the FFF:fff dimer that leads it to precipitate from water before it can mature into an extended fibrillary rippled sheet. Systematic optimization of crystallization parameters, including concentration, solvent identity, temperature, as well as variations in sequence, may allow the synthesis of extended fibrillary rippled sheet networks in the future. In that context it is interesting to compare our FFF:fff dimer structure with (a) the racemic Aβ40 structure, published in a recent collaborative study by the Raskatov and Tycko labs,^29^ and (b) the hydrophobic Aβ16–22 segment in its interactions with its mirror-image, studied by the Nilsson lab. All three systems contain rippled antiparallel dimers, which is likely due, at least in part, to coulombic attractions. However, there are important differences. Racemic Aβ40 forms fibrils with three Aβ40 units per layer and a fibril thickness of 7 ± 1 nm.^8^ The crystalline Aβ16–22 aggregates, on the other hand, are micron-wide, which is consistent with the presence of thousands of peptides per layer.^25^ Future X-ray structural studies of racemic Aβ16–22 should determine whether it (a) forms extended rippled sheets, (b) aggregates into rippled antiparallel cross-β dimers that then pack in ways similar to FFF:fff, or (c) packs in a way that is completely different.

Our findings have to be put in context with the recent paper by Liu and Gellman, where peptides designed to form two-stranded β-hairpins, composed of half L and half D residues did not exhibit any heterochiral stand pairing detectable by solution NMR.^24^ It is noteworthy that one of the systems studied by the authors contained the VFF motif that is present in Aβ and is believed to be important for racemic Aβ fibrillization (*i.e.*, Aβ Chiral Inactivation, Aβ-CI).^7,25,29^ The VFF motif is also very similar in terms of its size and hydrophobicity to the FFF motif studied here. A possible reason for the apparent discrepancy is that in Gellman's work, the L- and D- sequences were linked together, which may have induced a preference for homochiral strand pairing. Possibly more significantly, FFF:fff crystallization (similarly to Aβ-CI and the racemic Aβ16–22 model system studied by Nilsson) appears to occur under kinetic control, whereas the foldamers of the Gellman hairpin were monitored under thermodynamic equilibrium conditions. Similarly (albeit in the non-polar solvent CDCl_3_), Chung and Nowick found that hydrophobic β-turn peptide mimics preferentially form homochiral (pleated) dimers.^23^ Another important difference between our work and the two solution NMR studies is that, in our study, the rippled antiparallel FFF:fff dimers are packed into a three-dimensional crystal lattice that may, in itself, be a ripple-genic factor. In contrast, the solution NMR studies lacked evidence for the formation of higher order aggregates, and instead highlighted interactions between dimerizing peptide strands as isolated entities.

It may be tempting to ascribe the difference between the solution NMR experiments discussed above and our findings to the fact that solution NMR work studied systems as pure dimers, whereas our work produced extended layers, in which the individual dimers were stabilized through interactions with the crystal lattice. However, we are aware of a crystal structure of the GSTSTA peptide in a racemic mixture with its enantiomer, in which self-sorting into pleated fibrillary structures was observed, showing that racemic aggregating peptide mixtures are not ripple-genic *per se* either.^30^ In this specific case, it may have been because GSTSTA lacks bulky, hydrophobic groups that appear to promote rippled sheet formation.^17^ Yet it seems that the presence of bulky residues is not obligate either, as the first rippled sheet structure was reported for polyglycine I, which does not have sidechains.^18,19^ It should also be noted that, in addition to sequence, aggregation conditions are important. As such, it was noted with the MAX1:DMAX system developed by the Schneider lab, that the rigidity of the hydrogels formed depended on whether peptides were aggregated under kinetic or thermodynamic control, with thermodynamically controlled assembly producing the most rigid hydrogel systems.^3,4,17^ These are all conditions that should be explored in future research.

## Conclusions

We presented crystal-structural insights into a rippled sheet-based nanostructure that we obtained by temperature-controlled crystallization of FFF:fff. The structure consists of arrays of dimeric antiparallel rippled sheet, whose internal structural parameters agree well with the predictions by Pauling and Corey. The rippled dimers are arranged in a herringbone-pattern, into networks that are held together by in-plane salt bridges and hydrogen bonds and display lateral long-range segregation into hydrophobic and hydrophilic domains. Comparison of FFF:fff with the three orphaned rippled sheets identified by analyzing the racemic protein crystallography PDB supports the notion of Phe as a ripple-genic residue. Systematic exploration of Phe-containing racemic peptide mixtures may provide a rational framework on how to devise functional rippled sheet materials in the future.

## Materials and methods

### Peptide synthesis

The (l,l,l)-triphenylalanine (*i.e.*, FFF) and (d,d,d)-triphenylalanine (*i.e.*, fff) peptides were synthesized by standard Fmoc-based, solid-phase peptide chemistry, following our previously reported protocols.^39,51^ Both peptides were synthesized using preloaded, Fmoc-phenylalanine 4-alkoxybenzyl alcohol Wang resin:Fmoc-L-Phe-Wang (Sigma) or Fmoc-D-Phe-Wang (Fisher). All syntheses were performed manually at 0.2 mM scale relative to resin loading. An orbital shaker was used for mixing in both the deprotection and coupling steps. The resin was swelled in 3 mL of dimethylformamide (DMF) in a filter tube, housing 250 mg Fmoc-Phe Wang resin (0.796 mmol g^−1^ loading) for 20 min. For Fmoc-deprotection, 30% piperidine (spectrum) in DMF was added to the resin, and allowed to shake on an orbital shaker for 20 min. The deprotection solution was rinsed with DMF (3×) and dichloromethane (DCM, 2×) and the deprotection step was repeated. Coupling reagents used were 4 eq. *N*,*N*-diisopropylethylamine (Fisher), 3 eq. *N*,*N*,*N*′,*N*′-tetramethyl-*O*-(1*H*-benzotriazol-1-yl)uronium hexafluorophosphate (Fisher) and 3 eq. hydroxybenzotriazole hydrate (Oakwood Products). For amino acid coupling, 3 eq. of either Fmoc-L-Phe-OH (Fisher) or Fmoc-D-Phe-OH (ChemPep) with coupling reagents listed above were dissolved in 3 mL DMF and added to the reaction vessel, and allowed to shake for 30 min. The coupling step was repeated for each amino acid addition to improve yield. The aforementioned steps were repeated to produce the resin-bound tripeptides, NH_2_-L-FFF-COOH and NH_2_-D-fff-COOH. The peptides were cleaved and deprotected with a mixture consisting of trifluoroacetic acid (10 mL, Fisher), tri-isopropylsilane (1 mL, Fisher), and liquefied phenol (0.5 mL, Sigma). The peptide identities were confirmed with mass spectrometry (Fig. S1 and S2‡). Peptides were purified by reverse-phase high-performance liquid chromatography (HPLC) with PLRP-S columns (Agilent), as previously described,^39,51^ yielding peptides with purities exceeding 95% (Fig. S1 and S2‡). HPLC was conducted under basic conditions (0.1% NH_4_OH), to reduce aggregation and/or precipitation. Samples were lyophilized and stored as solid powders at −40 °C.

### Crystallization

Solutions of L-FFF and D-fff peptides were prepared separately by dissolving 7 mg of each individual peptide in 4 mL of nanopure water. The resulting solutions were sonicated and transferred to an oil bath at 90 °C and kept under stirring for one hour. To enhance dissolution of the cloudy slurries, 80 μL of hexafluoroisopropanol (HFIP; Fisher) was added to the solutions (2% of total volume), but significant cloudiness was still observed. After an additional 1 h of heating in the oil bath, the two individual peptide solutions were combined by adding D-fff to the L-FFF solution, dropwise. The resulting cloudy solution was rapidly transferred to a Teflon lined stainless steel autoclave, which was sealed and placed on an oven at 75 °C for 10 days followed by a slow colling process at a rate of 0.1 °C min^−1^, leading to the formation of colorless, needle-like crystals.

### Single-crystal X-ray diffraction

A suitable colorless needle with dimensions of 0.1 × 0.09 × 0.03 mm^3^ was used for single-crystal X-ray diffraction data collection at 100 K on a Rigaku XtaLAB Synergy-S diffractometer using Cu K_α_ radiation (*λ* = 1.54 Å). Data collection, processing and reduction were performed with CrysAlis^Pro^.^52^ After face indexing, numerical absorption correction was applied using Gaussian integration. Empirical absorption correction using spherical harmonics was applied using SCALE3 ABSPACK scaling algorithm. The structure was solved by intrinsic phasing using ShelXT and refined with ShelXL *via* Olex2.^53–55^ All non-hydrogen atoms were refined anisotropically using standard procedures.^56^ Atomic displacement parameters for hydrogen atoms in the terminal amine group were fixed to 1.5(*U*_iso_) of the attached nitrogen atom. For all other hydrogen atoms, the values were fixed to 1.2(*U*_iso_) of the atoms to which they are attached. The N–H distances in the amine and amide groups were restrained to 0.91(2) Å and 0.88(2) Å, respectively. All other hydrogen atoms were placed at geometrically calculated positions and refined using a riding model.

### Computational chemistry

The input geometry for the optimization of FFF:fff was generated using the crystallographic data. The optimization was performed using ORCA 4.2.1, using Becke's 1988 exchange functional and Perdew's 1986 correlation functional (*i.e.*, BP86)^57,58^ and the resolution of the identity approximation. Ahlrichs' def2-SVP basis set and the def2/J auxiliary basis set were used.^59,60^ An atom-pairwise dispersion correction with the Becke–Johnson damping scheme was applied (D3BJ).^61,62^ Implicit aqueous solvation was achieved using a conductor-like polarizable continuum model (CPCM = water).^63^

### CSD search

A systematic search of the CSD (version 5.41) was performed using ConQuest (version 2.0.4). Two queries were submitted simultaneously. The first searched for a C(C)C(O)NHC(C)C(O)NHC(C)C(O)NH fragment with all bond types set to “any”, with both *φ* torsion angles from -180–0°, and with both *ψ* torsion angles within the range 0–180°. The second query required the presence of a distinct C(C)C(O)NHC(C)C(O)NHC(C)C(O)NH fragment with all bond types set to “any”, with both *φ* torsion angles from 0–180°, and with both *ψ* torsion angles within the range −180–0°. The hits from this search were inspected manually and none featured a rippled sheet motif.

### PDB structural database mining

The PDB database was searched for the term “Racemic”, and the results were narrowed by selecting “protein” as the polymer entity type, producing a total of 387 hits. The majority of those hits were, however, not truly racemic protein structures, but rather, enantiomerically pure proteins complexed with racemic molecules or simply included racemic compounds used during synthesis. These were excluded from our search. From the remaining hits, we manually selected those, in which the mirror-image proteins had β-strands oriented in ways that made them potentially capable of forming rippled sheets. This eventually produced three structures that can be accessed through the PDB *via* reference codes 4WPY,^42^4IUZ,^43^ and 3ODV.^44^

### Considerations regarding nomenclature

In the original theory papers Pauling and Corey introduced the concepts of the pleated sheet that since became textbook knowledge as the β-sheet, and the closely related, but understudied rippled sheet.^16^ Those seminal papers discussed periodic layer structures, and the original definition of sheets originated from there. However, this nomenclature since evolved: it is now common to refer to adequately paired peptide strands of the same handedness as pleated β-sheets. In this paper we follow analogy and refer to adequately paired peptide strands of opposite chirality as rippled β-sheets. The periodic β-sheets are discussed in the context of fibril structures, which is specified where necessary.

## Data availability

Crystal structural data are available *via* CCDC 2124137.

## Author contributions

Conceptualization: J. A. R. Investigation, formal analysis, and methodology: A. J. K., B. E., J. A. R., and T. C. J. Supervision, resources, and funding acquisition: J. A. R., S. R. J. O., and T. C. J. Writing – original draft: A. J. K. and J. A. R. Writing – review and editing: all authors.

## Conflicts of interest

There are no conflicts to declare.

## Supplementary Material

SC-013-D1SC05731F-s001

SC-013-D1SC05731F-s002
