# Supplementary material for: A crystal-structural study of Pauling–Corey rippled sheets
Source: Chem Sci. 2021 Dec 8;13(3):671–80. doi: 10.1039/d1sc05731f (PMC8768883; doi:10.1039/d1sc05731f)
Supplement: SC-013-D1SC05731F-s001 [file SC-013-D1SC05731F-s001.pdf]

Electronic Supplementary Material (ESI) for Chemical Science.
This journal is © The Royal Society of Chemistry 2021

Supplementary Information for
A Crystal-Structural Study of Pauling-Corey Rippled Sheets

Ariel J. Kuhn,[a,♪] Beatriz Ehlke,[a,♪] Timothy C. Johnstone,[a]
Scott R. J. Oliver,[a] Jevgenij A. Raskatov[a,*]

* corresponding author; ♪ denotes equal contribution

Email: jraskato@ucsc.edu

This PDF file includes:

Figures S1 to S8
Tables S1 to S2
References

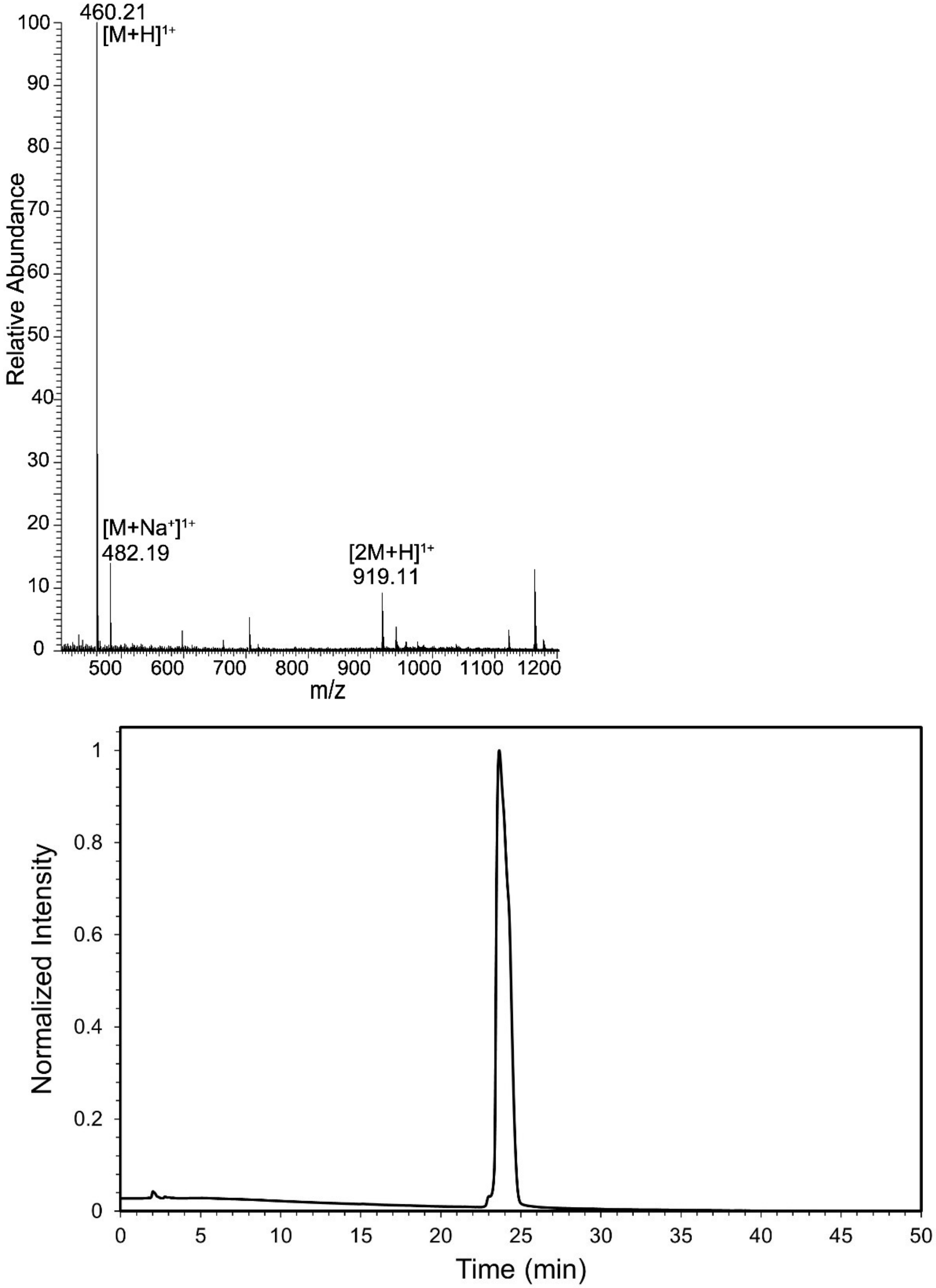

Fig. S1. Mass Spectrometry and HPLC characterization for NH_2-L-FFF-COOH.

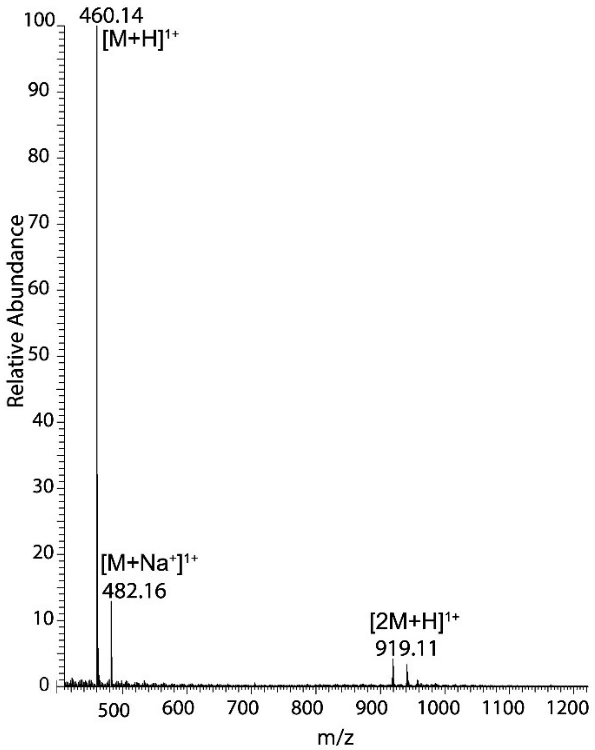

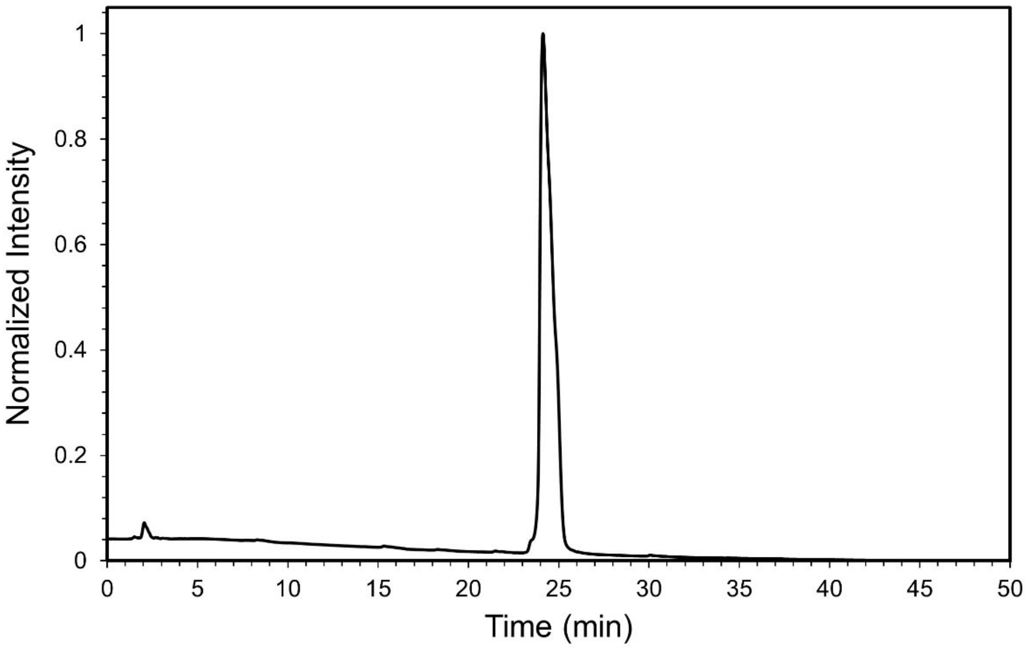

Fig. S2. Mass Spectrometry and HPLC characterization for NH_2-D-FFF-COOH.

Table S1. Crystallographic parameters

Compound	Racemic triphenylalanine
Formula	$C_{27}H_{29}N_3O_4$
FW	459.53
T (K)	100(2)
λ (Å)	1.54184
Crystal System	Monoclinic
Space group	$P2_1/c$
a (Å)	11.3563(5)
b (Å)	16.7472(6)
c (Å)	12.6545(6)
β (°)	101.483(4)
Volume (Å^3)	2358.54(18)
Z	4
ρ_{calc} (Mg/m^3)	1.294
Size (mm^3)	0.10 × 0.09 × 0.03
θ range (°)	4.44 – 67.06
Total data	23314
Unique data	4187
Parameters	323
Completeness	99.7%
R_{int}	5.00%
R_1 (I > 2σ)	4.43%
R_1 (all data)	5.54%
wR_2 (I > 2σ)	11.22%
wR_2 (all data)	11.81%
S	1.036
Min, max (e Å^{-3})	–0.279, 0.295

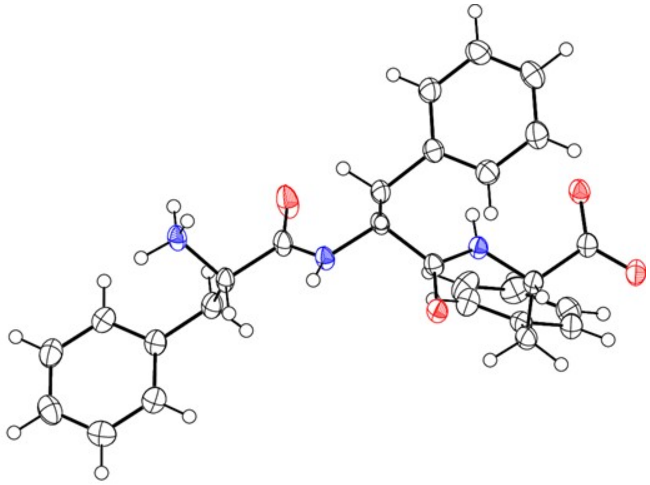

Fig. S3. Molecular graph of FFF with 50% thermal ellipsoids and H atoms shown as spheres of arbitrary radius. Color code: O red, N blue, C grey, H white.

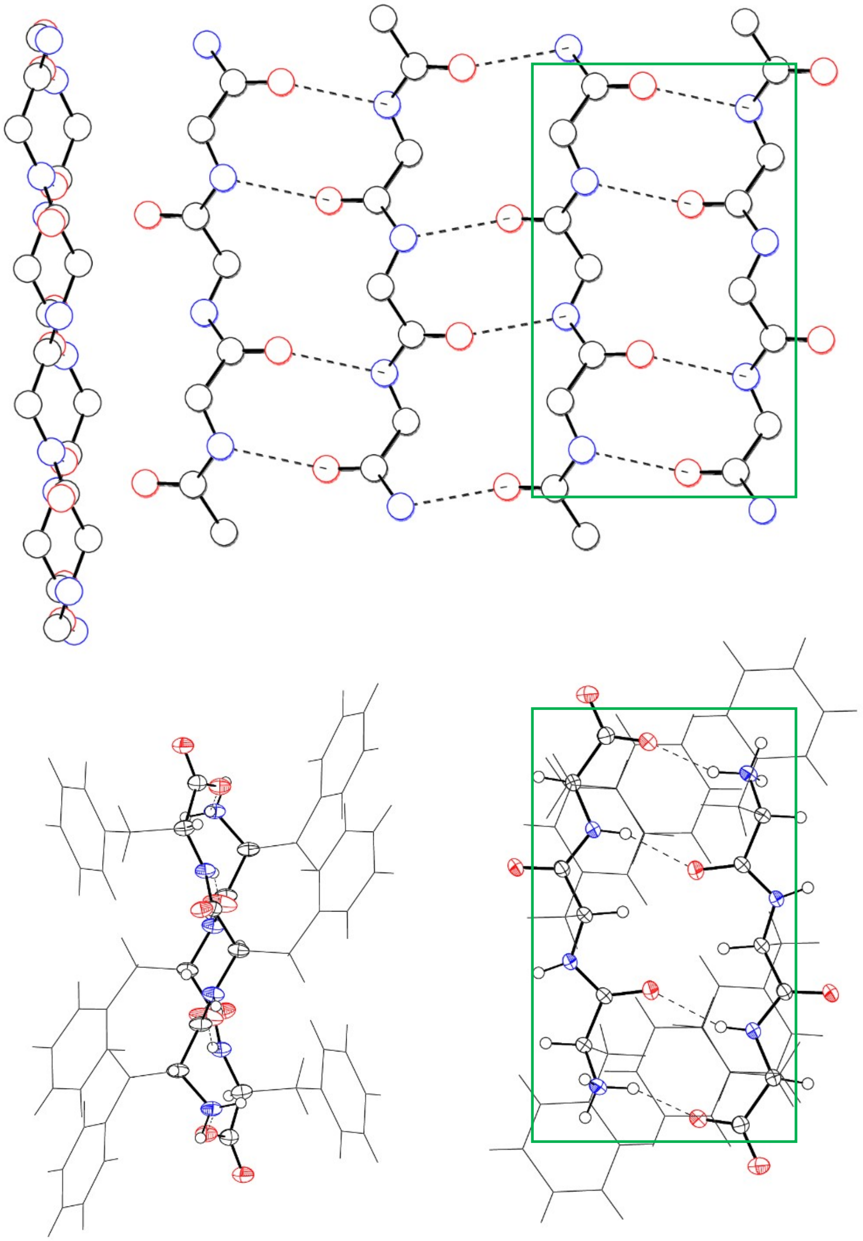

Fig. S4. _Top:_ Side and top views of the rippled antiparallel sheet structure predicted by Pauling and Corey [cf. Figure 1 from *PNAS* **1953**, *39*, 253]. _Bottom:_ Side and top views of the rippled sheet structure formed by FFF:fff. Atoms of the polypeptide backbone are shown as ellipsoids (non-H) or spheres of arbitrary radius (H). Side-chain atoms are shown as sticks. Color code: O red, N blue, C grey, H white. Hydrogen bonds are shown as dashed lines. Boxed in green is the similar motif from the predicted (top) and experimentally observed (bottom) structure.

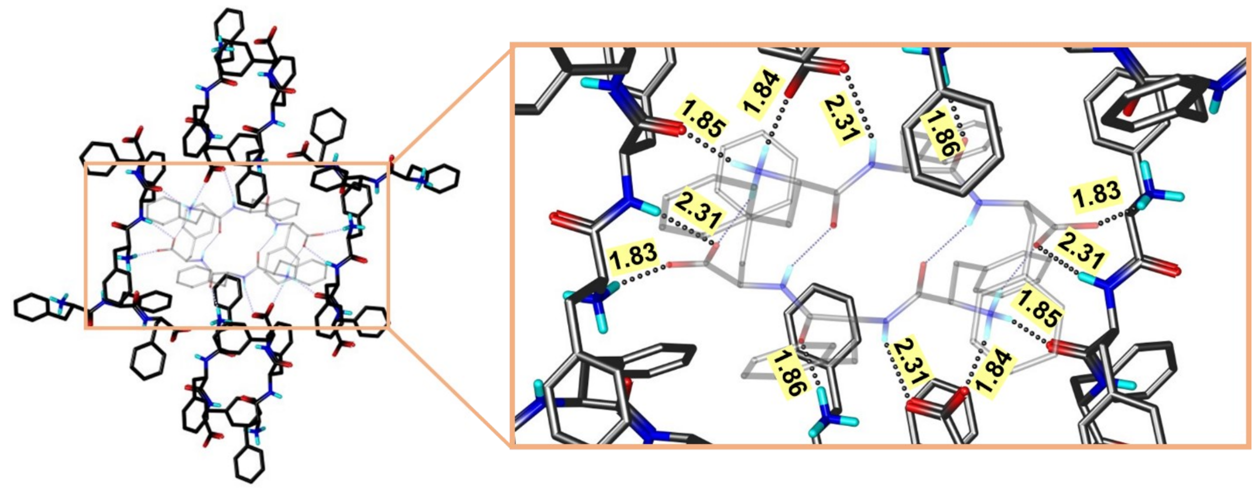

Fig. S5. The rippled antiparallel FFF:fff cross-β dimer shown in contact with its direct neighbors.

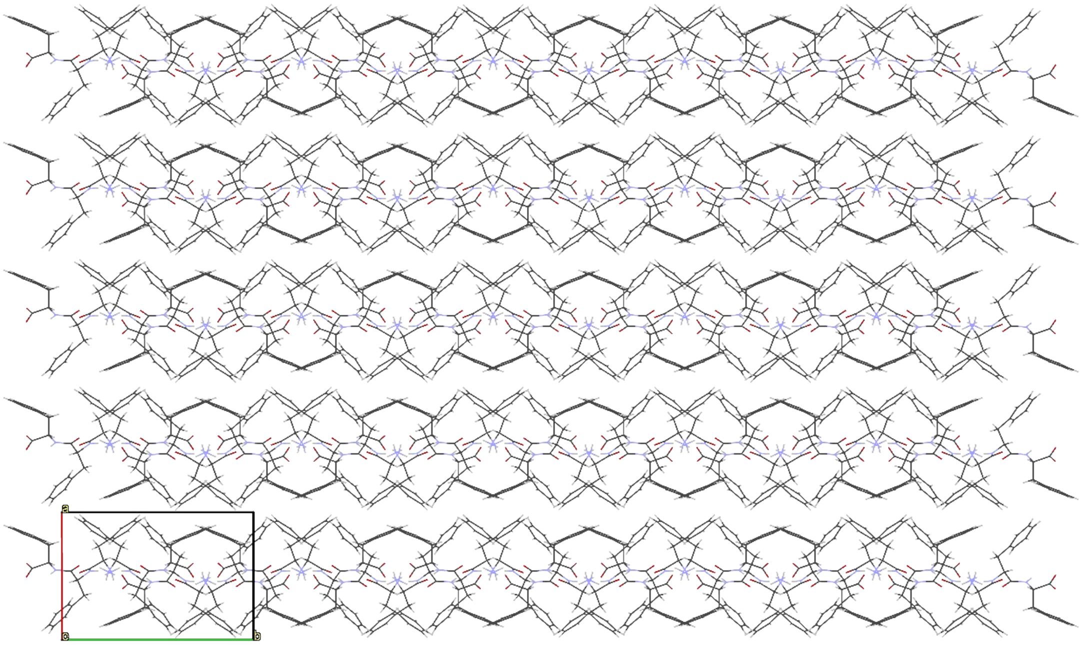

Fig. S6. Packing diagram of FFF:fff highlighting that the layer-to-layer distance is the crystallographic lattice parameter *a* = 11.3563(5) Å.

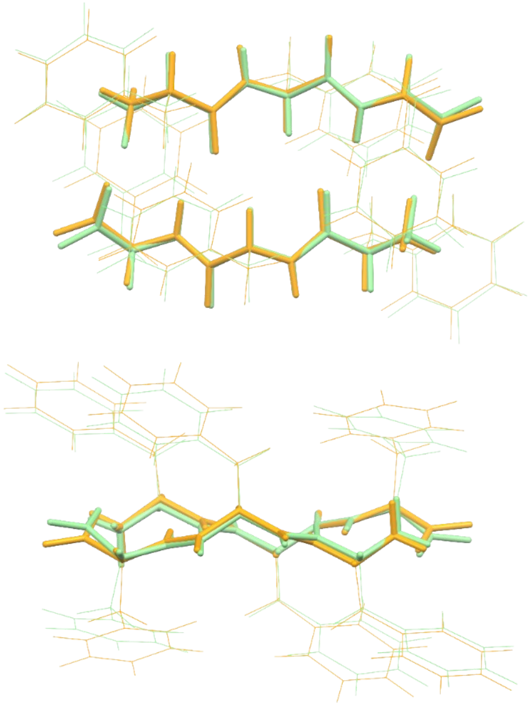

Fig. S7. Overlay of the crystallographic (orange) and the optimized (green; BP86-D3BJ/def2-SVP-CPCM$_{water}$) structure of the FFF:fff dimer. The polypeptide backbone is shown as thick sticks and the sidechains as thin sticks.

Table S2. Cartesian coordinates (Å) of the optimized (BP86-D3BJ/def2-SVP-CPCM$_{water}$) structure of FFF:fff.

N	4.09370415118074	12.55158927464286	8.01951364412157
H	3.96372050731073	11.87945342785070	8.89432214522468
H	3.67631800795364	13.45649622333425	8.29311390077636
H	5.10684826815001	12.72465726857804	7.86526886151337
O	4.68579699986856	10.00514061707158	7.08428367684645
C	4.21448263423020	10.83662052651225	6.28704928091444
C	3.40980475436177	12.02854135266891	6.80005942484666
H	3.38849381951550	12.83743527898451	6.04322345172614
C	1.97814220013488	11.57525758215830	7.15655122642748
H	1.54884004221062	11.10109355234939	6.25346523955252
H	2.05396185690734	10.79648718941633	7.94155838244508
C	1.09701622979355	12.71237139894564	7.62055690280438
C	1.09001748865928	13.10851995683912	8.97692250143198
H	1.71128992818933	12.55279025588311	9.70001424570751
C	0.29689600869346	14.19002383941204	9.39669431116368
H	0.30059192960416	14.48984062825498	10.45650801618807
C	-0.50386245035868	14.88372023555253	8.47211911233997
H	-1.12640255974232	15.72951255555404	8.80337394705965
C	-0.50629168506993	14.49105974185670	7.12126056936911
H	-1.13393642246107	15.02638237004613	6.39177196518855
C	0.29214352294006	13.41473994354018	6.69959318764820
H	0.28717212918944	13.10692663820845	5.64159702917600
N	4.34294104144003	10.71642127776430	4.95139847892585
H	4.02617325620660	11.45980460038944	4.32051429068197
O	4.39183936657579	10.13695537762992	2.13900493459608
C	4.53310543154521	9.25649096794045	3.00946511222600
C	5.11461928813669	9.61794373703439	4.38145018136459
H	5.03142864014147	8.76479723071096	5.07907335395468
C	6.60638929528331	10.03204527673807	4.24216281816270
H	6.66557687589099	10.81001469482846	3.45165198777844
H	6.89946224238773	10.50545984207140	5.19887788497200
C	7.54254308228714	8.88459583473587	3.94395032674684
C	7.58844472576498	8.28365432032168	2.66674556186903
H	6.94541458912158	8.66743223090499	1.86030598840700
C	8.45406241058803	7.20893971981611	2.40909750515203
H	8.45866869918136	6.74144750394299	1.41303033853607
C	9.30756466458947	6.72918695577871	3.41817773094835
H	9.98108783486585	5.88275984854788	3.21748271978528
C	9.27910482335856	7.32382914438217	4.69135159978216
H	9.93367021228568	6.94739162654751	5.49237800535179
C	8.39410679692009	8.38361619037178	4.95215142881515
H	8.36419766813895	8.83646995965917	5.95638546896319
N	4.27663118712145	7.94432643547798	2.83632745682123
H	4.39016191615368	7.30690269470780	3.64608983816175
O	5.36980195556720	5.51211468406074	2.26698208562355
O	5.09304882803972	5.85257505134016	0.05486234185960
C	4.90057255794537	6.14531097208455	1.25187163274656
C	3.97434193707562	7.35780504501355	1.54693871462719
H	4.15988268217939	8.13603868377272	0.78141118168691
C	2.48655619546023	6.92138841586021	1.42477292423107
H	1.85209546270454	7.81224356928512	1.60606231857112
H	2.32724214528117	6.59796256665133	0.37609533953726
C	2.11097727051036	5.80620589219198	2.37244680421324

C	2.28383758162549	4.45495364572536	1.99379188158820
H	2.67080634096225	4.22534617366592	0.98911503166958
C	1.97216973402590	3.41055767808345	2.87910349859398
H	2.11294850143188	2.36460126016809	2.56553803860911
C	1.48716530888976	3.70102699174574	4.17042290894159
H	1.23529636180071	2.88618588539751	4.86589264946698
C	1.31363145278839	5.04280868136374	4.55795607578832
H	0.92521192817041	5.27458827729304	5.56116588140656
C	1.62688866751194	6.08414190438623	3.66815534639682
H	1.50038556562449	7.13045249278436	3.98520466935573
N	4.74703122774710	4.19342206535575	4.38663953239223
H	4.86982114067797	4.86859999022297	3.51236735404479
H	5.16572951866549	3.29077403479728	4.10731414188434
H	3.73588967731612	4.01485485660771	4.54727049323470
O	4.16396127954101	6.74099311505567	5.31970178886795
C	4.63668287937818	5.91001586461808	6.11657509188658
C	5.43805826522110	4.71658749571353	5.60216894786604
H	5.46305755532070	3.90877021067308	6.35997761795219
C	6.86792610873869	5.16821719136807	5.23686214111084
H	7.30324336496732	5.64298378557832	6.13686772890228
H	6.78863229433642	5.94642718974931	4.45160130783574
C	7.74531512969249	4.02995894798071	4.76861216913461
C	7.74620225810931	3.63468321238378	3.41199288294599
H	7.12226633738760	4.19134827437473	2.69196386862010
C	8.53610557380459	2.55234105284025	2.98827944757836
H	8.52777712362381	2.25340164652189	1.92824534422122
C	9.33923708665485	1.85655064983315	3.90916358454985
H	9.95906506252588	1.00998479980240	3.57486101451993
C	9.34747886624511	2.24803499228983	5.26035139643555
H	9.97688767485362	1.71100378114162	5.98706279816186
C	8.55257204045476	3.32541707508023	5.68587348668957
H	8.56224206367965	3.63190721945685	6.74422251177071
N	4.51052720974095	6.03229462726670	7.45220154621982
H	4.82651042328429	5.28942368673009	8.08403981081477
O	4.45232595927066	6.60889428488492	10.26408493469117
C	4.30897502190871	7.49022987865021	9.39504697506979
C	3.73597511114322	7.13021966374973	8.01935926195330
H	3.82437115588301	7.98469998990265	7.32401109400939
C	2.24330073099328	6.71538912344956	8.14594351566544
H	2.17747246094972	5.93349904559341	8.93207273703793
H	1.95932459305754	6.24640532392327	7.18443283902556
C	1.30292448053796	7.86019962901400	8.44063142782111
C	1.24628926054932	8.45719361506985	9.71925040002921
H	1.88425058064177	8.07248796859544	10.52931357266964
C	0.37671889880669	9.52946860213518	9.97359028393214
H	0.36398847124617	9.99405460084568	10.97094721913926
C	-0.47041059278953	10.01046241701172	8.95970510299747
H	-1.14694010497439	10.85507471550927	9.15804996991875
C	-0.43155406803901	9.41947292909609	7.68513411129953
H	-1.08076873075477	9.79703992510457	6.88030795999379
C	0.45762561067833	8.36233045564615	7.42775676507842
H	0.49635272347544	7.91346605546526	6.42204849777097
N	4.55794847339994	8.80345073438008	9.57209305360787
H	4.44090072447028	9.44241168791802	8.76417612467272
O	3.45773153209174	11.23195820553672	10.13619692761770
O	3.72372766929813	10.89370536486153	12.35022202628192

C	3.92218845055026	10.60018630256060	11.15420196520344
C	4.85142631989578	9.38888224494806	10.86400355769733
H	4.66304253740939	8.60924727106737	11.62747022693811
C	6.33799403486710	9.82787788232499	10.99427526631197
H	6.97470662291954	8.93916325253939	10.81038900872820
H	6.49410552636178	10.14606052384495	12.04506325579148
C	6.71402078474572	10.94844574207184	10.05328804868640
C	6.54366053238547	12.29762245682966	10.44049823518437
H	6.15815230682130	12.52157842727393	11.44705665043399
C	6.85585320690155	13.34711395800373	9.56158813110815
H	6.71736316114667	14.39123957898378	9.88217394534763
C	7.33815102249408	13.06412388275726	8.26764787422629
H	7.58955393048688	13.88299875538902	7.57680417956278
C	7.50710227758705	11.72451427242645	7.87058479718694
H	7.88968207936726	11.49728715754806	6.86407916177420
C	7.19436456586794	10.67803224808990	8.75476176529525
H	7.31699362953703	9.63366286271353	8.42976376320827

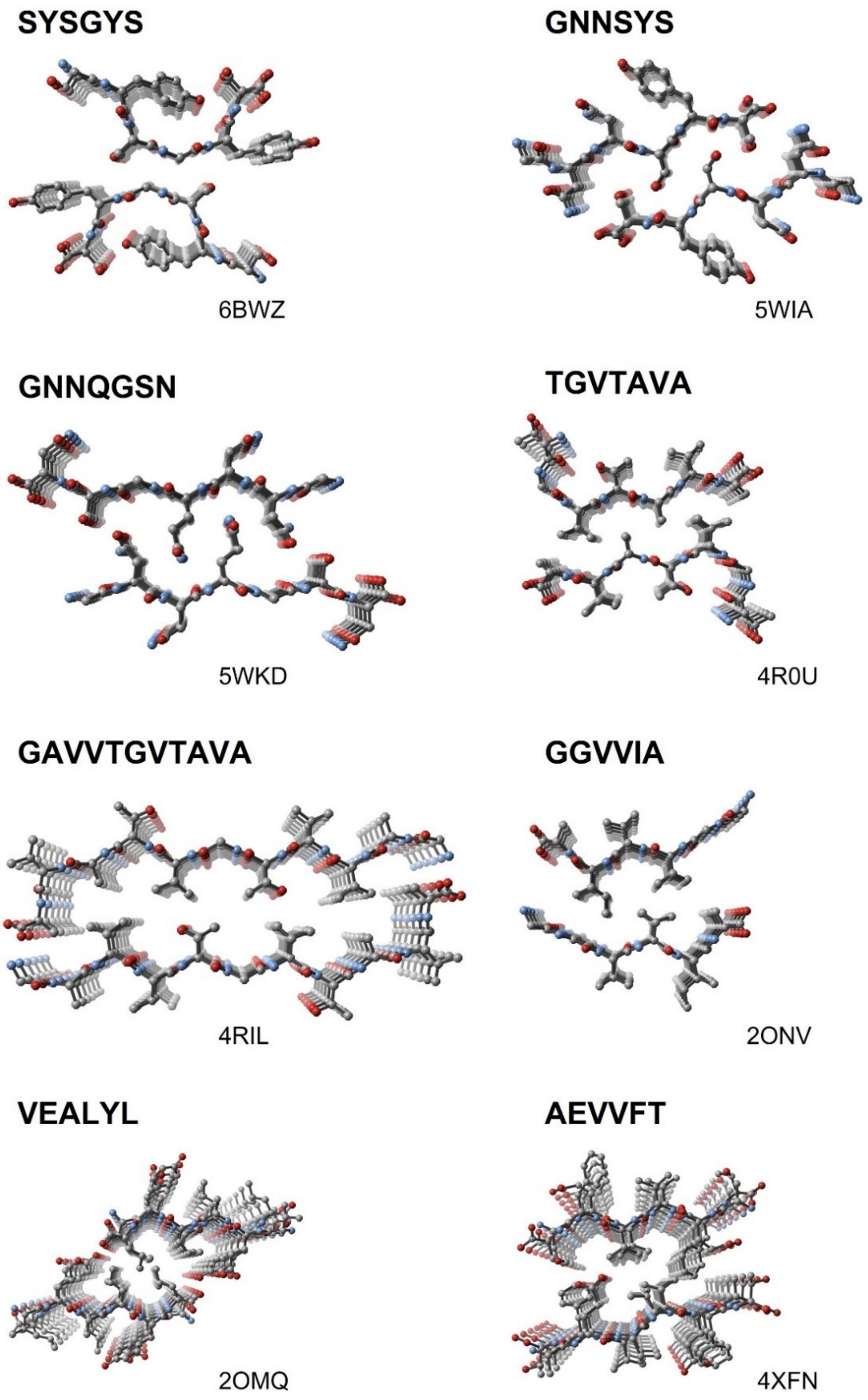

Fig. S8. Selected examples from Eisenberg and coworkers,(1–6) of fibrils formed by aggregating enantiopure peptides in which pleated β-sheets display one-dimensional, long-range order. Color code: C, gray; O, red; N, blue. Water molecules and hydrogens are omitted for clarity. PDB codes are displayed on the bottom right of each structure.

REFERENCES

1. M. P. Hughes, *et al.*, Atomic structures of low-complexity protein segments reveal kinked β sheets that assemble networks. *Science* **359**, 698-701 (2018).

2. E. L. Guenther, *et al.*, Atomic structures of TDP-43 LCD segments and insights into reversible or pathogenic aggregation. *Nat. Struct. Mol. Biol.* **25**, 463–471 (2018).

3. D. Li, *et al.*, Structure-Based Design of Functional Amyloid Materials. *J. Am. Chem. Soc.* **136**, 18044–18051 (2014).

4. J. A. Rodriguez, *et al.*, Structure of the toxic core of α-synuclein from invisible crystals. *Nature* **525**, 486–490 (2015).

5. M. R. Sawaya, *et al.*, Atomic structures of amyloid cross-β spines reveal varied steric zippers. *Nature* **447**, 453–457 (2007).

6. L. Saelices, *et al.*, Uncovering the Mechanism of Aggregation of Human Transthyretin. *J. Biol. Chem.* **290**, 28932–28943 (2015).
